# Metastatic Gastric Carcinoma Presenting as Acute Appendicitis

**DOI:** 10.7759/cureus.7617

**Published:** 2020-04-10

**Authors:** Saad Siddiqui, Muhammad Yaseen, Kashif Sajjad

**Affiliations:** 1 Radiology, Shaukat Khanum Memorial Hospital & Research Centre, Peshawar, PAK; 2 Internal Medicine, Shaukat Khanum Memorial Hospital & Research Centre, Peshawar, PAK

**Keywords:** acute appendicitis, metastasis, acute abdomen, computed tomography

## Abstract

Metastatic disease is one of the few rare causes which can present with clinical and radiological features of acute appendicitis. In this article, we present a case of a 33-years-old man with known primary gastric malignancy undergoing adjuvant treatment, who presented with clinical peritonitis. Imaging findings revealed acute appendicitis and a sealed-off appendiceal perforation. Diagnosis of metastatic adenocarcinoma to the appendix was confirmed on histopathology. Sound knowledge among clinicians and radiologists regarding clinical presentation and radiological findings of acute abdomen in patients with known primary malignancy can aid in rapid diagnosis and management.

## Introduction

Metastatic disease to the appendix is a rare entity, the first presentation of which is usually acute appendicitis. Contrast-enhanced computed tomography (CT) is the modality of choice and usually reveals signs of appendiceal inflammation with a dilated caliber of the appendix, luminal obliteration by fluid, peri-appendiceal inflammatory changes as well as free and loculated fluid collections [1-3].

We report the case of a 33-year-old man presenting with acute appendicitis resulting in sealed off appendiceal perforation secondary to metastatic adenocarcinoma from primary gastric cancer which was subsequently confirmed upon histopathology.

## Case presentation

A 33-year-old man who had undergone partial gastrectomy for gastric adenocarcinoma was undergoing adjuvant chemotherapy. His disease was in remission with no evidence of residual disease or recurrence on surveillance post-operative scans and endoscopic examinations. He presented to the emergency room two months after the start of adjuvant treatment with complaints of severe diffuse abdominal pain having a severity of 10/10 on the numeric pain intensity scale. His body mass index (BMI) was 24.5 kg/m2. His vital signs revealed tachycardia with a pulse rate of 118 beats per minute, fever of 38 °C, blood pressure of 120/80 mmHg, respiratory rate of 19 per minute, and oxygen saturation of 96% on room air. General and systemic examination was positive for generalized distress and severe diffuse abdominal tenderness upon palpation. Initial lab investigations revealed markedly increased white cell count of 23000/microliter and significantly increased serum lactate level of 43 mg/dL.

He was admitted to the high dependency unit and started on supportive therapy, antibiotics, and analgesia. An urgent CT scan of the abdomen was ordered to determine the cause of his abdominal pain.

**Figure 1:**
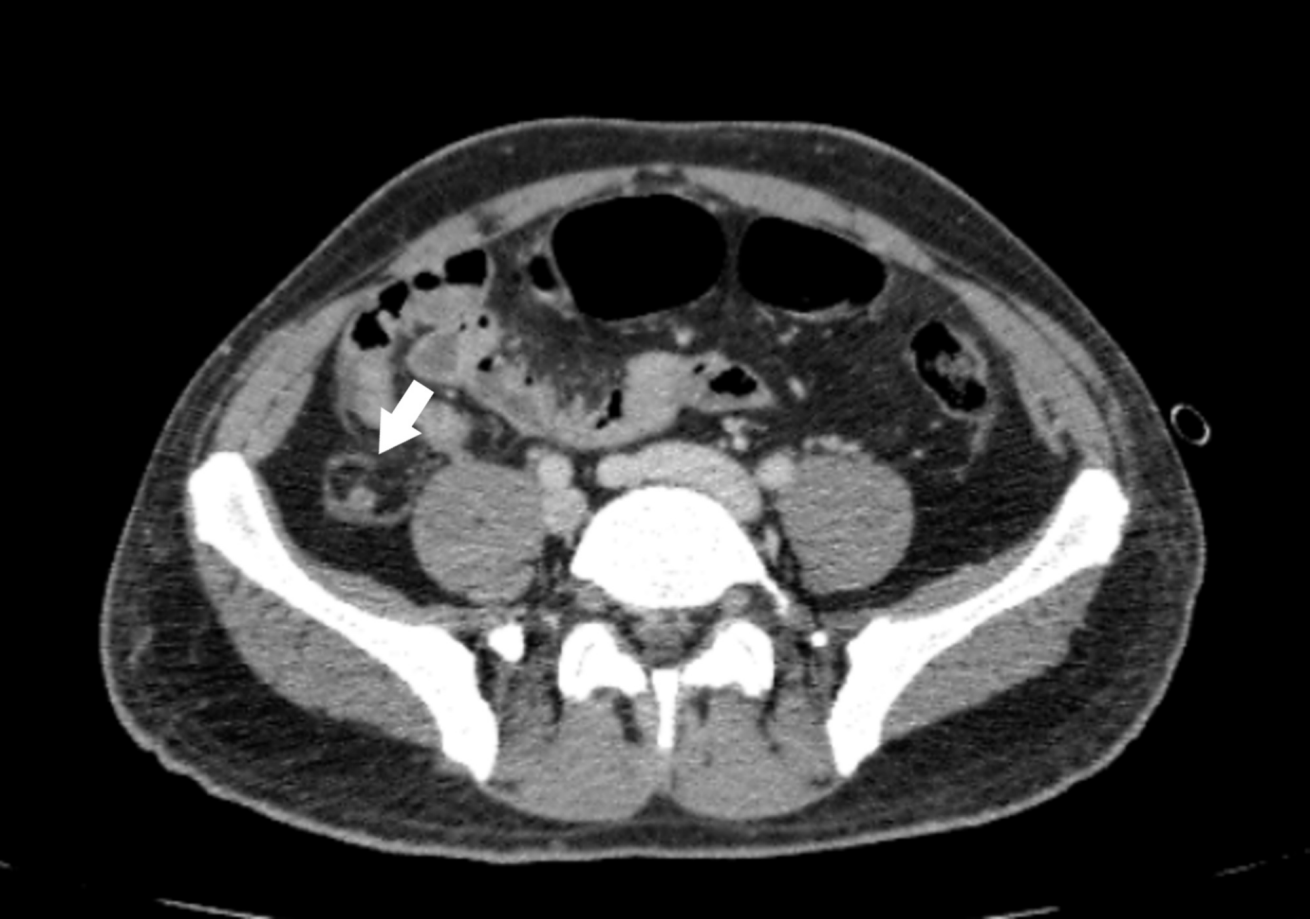
Dilated fluid-filled appendix with peri appendiceal inflammatory changes

**Figure 2:**
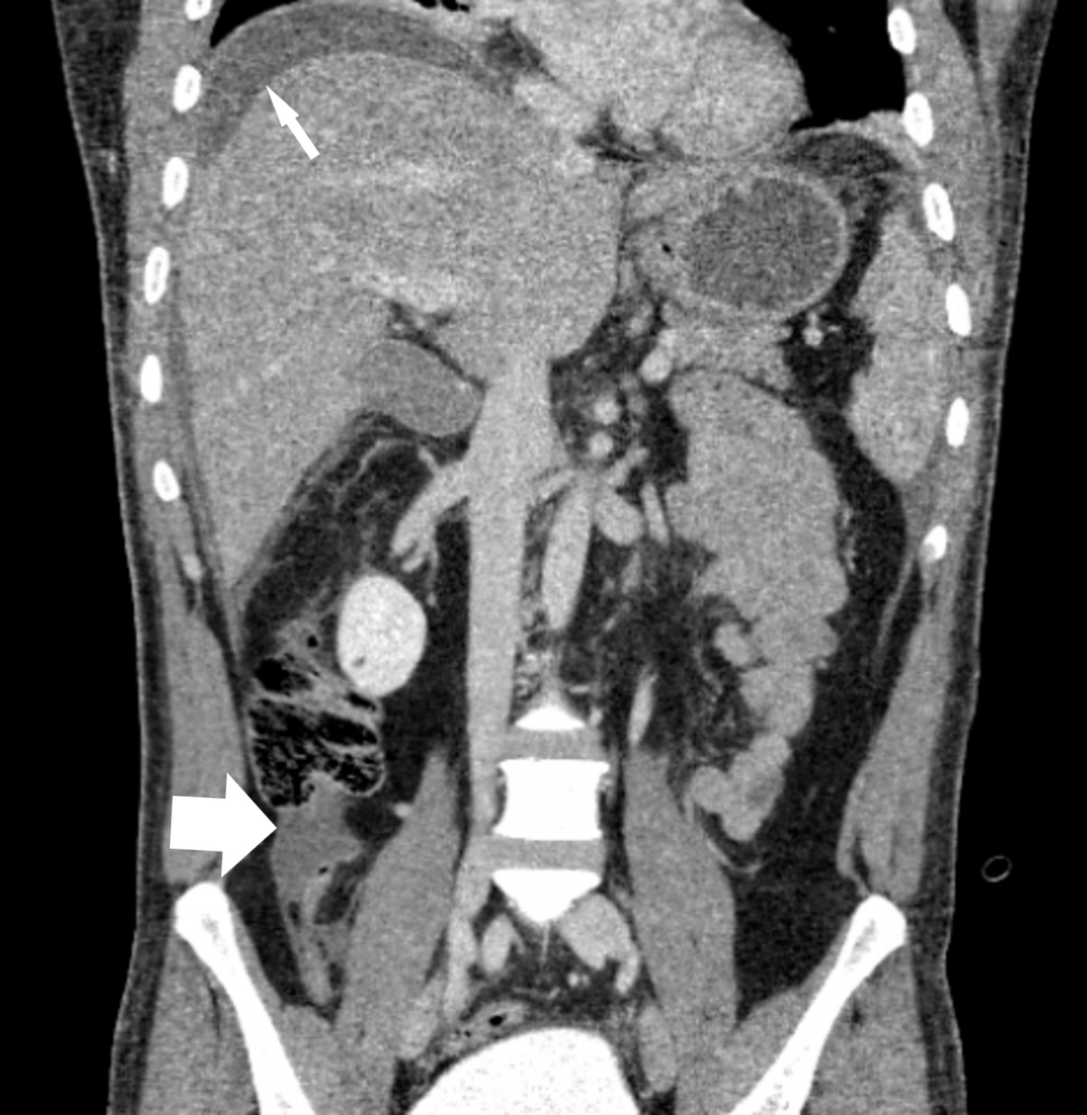
Perihepatic ascites (thin arrow); dilated appendix and peri appendiceal fluid collection is also noted (bold arrow)

A contrast-enhanced CT of the abdomen was subsequently performed to evaluate the cause of abdominal tenderness. There was no evidence of local disease recurrence on the scan. However, the appendix was dilated in caliber measuring up to 10 mm. It had surrounding inflammatory changes with peri appendiceal localized fluid collection and fat stranding (Figures 1-2). Upon a detailed look using different windowing levels, a note was made of a focal defect in the appendiceal wall integrity which was not disrupted on the serosal aspect. The appendix was enhancing and contained some intraluminal fluid. There were moderate ascites as well. Considering the overall appearances, a diagnosis of sealed off appendiceal perforation was made.

The patient underwent an emergency laparotomy and appendectomy. Per-operatively the appendix was mildly inflamed and a large volume of ascites was drained. The anastomotic site of prior gastric surgery was unremarkable. He was shifted to the intensive care unit and mechanically ventilated for two days. His post-operative course was on a continuous improving trend and he was subsequently shifted to the general ward and later discharged from the hospital in a stable, asymptomatic condition. 

The histopathological examination of appendectomy specimen subsequently revealed inflammatory changes of the appendix. There were foci of poorly differentiated carcinoma as well. The immunohistochemical analysis was positive for cytokeratin and mucicarmine. A final diagnosis of metastatic adenocarcinoma with adjacent soft tissue involvement was made.

## Discussion

Metastatic disease to the appendix is sparsely reported in the literature with common primary sites usually being the gastrointestinal and urogenital tracts, breast, lung, and the gallbladder [1,3-6]. Metastatic involvement of the appendix by primary gastric tumor was first described in 1951 by Goldfarb and till date, only a limited number of cases have been reported [1-4]. The most common reported presentation is the development of acute appendicitis and associated peritonism, the same as in our case. All patients in the literature were treated with appendectomy and diagnosis was confirmed on subsequent histopathology [6]. The prognosis after the diagnosis of solitary appendiceal metastasis is still a matter of debate but fluorodeoxyglucose/positron emission tomography (FDG/PET) scans have an evolving role in assessing the disease status in these patients [3].

## Conclusions

Acute appendicitis can rarely be caused by metastatic spread to the appendix from a known primary malignancy. In this article, we present a case of a patient with known primary gastric adenocarcinoma who presented with signs and symptoms of acute abdomen; he was found to have acute appendicitis with a sealed-off perforation on imaging and the histopathology of appendectomy specimen revealed metastatic disease. There can be a number of reasons resulting in the possible delay in workup and treatment of this condition e.g. low clinical suspicion of appendicitis as a cause of acute abdomen and other mimickers which are more common in oncology patients like intestinal obstruction or bowel ischemia. Improving knowledge among clinicians and radiologists regarding this rare entity can help in rapid diagnosis and treatment.
